# The Effect of Contrast Bath Therapy and Knee Pad Device on Pain, Range of Motion, and Functional Disability in Patients With Osteoarthritis Knee: A Randomized Control Trial

**DOI:** 10.7759/cureus.47586

**Published:** 2023-10-24

**Authors:** Pranali Fokmare, Pratik Phansopkar

**Affiliations:** 1 Musculoskeletal Physiotherapy, Ravi Nair Physiotherapy College, Datta Meghe Institute of Higher Education & Research (Deemed to Be University), Wardha, IND; 2 Research & Development (R&D), Ravi Nair Physiotherapy College, Datta Meghe Institute of Higher Education & Research (Deemed to Be University), Wardha, IND

**Keywords:** vibration, quality of life, pain, heating, cryotherapy

## Abstract

Background and objective

A degenerative joint condition mostly affecting the weight-bearing joints is osteoarthritis (OA). The majority of the time, it involves the knee joint. Pain and stiffness are common in grade 1 and 2 OA. And that's the main reason people ask for help. Physiotherapy treatment can be helpful for symptomatic management of early OA. Along with exercises, contrast bath therapy (CBT) is a therapeutic alternative to medication to alleviate pain and stiffness in OA. Many studies have been done using the traditional water immersion CBT. However, there is a paucity of studies on contrast therapy given using a device. This study intends to find the effect of a knee pad device (KPD) on pain, range of motion, and functional disability in knee OA patients when compared with CBT.

Methods

About 60 patients having unilateral knee OA were selected and randomly divided into two groups: group A received CBT for 20 minutes, and group B was treated with a KPD for 20 minutes and the Otago exercise program was given in both groups for 30 minutes. Both groups received treatment for three sessions per week for two weeks. Outcome measures used for assessment at baseline and post-treatment were visual analog scale (VAS), knee range of motion, Western Ontario and McMaster Universities Arthritis Index (WOMAC) scale, and distance covered in a two-minute walk test.

Results

Both the groups showed significant improvement post-treatment (p < 0.05). Group B showed more significant improvement when compared with group A. The enhancement in VAS (2.39, p < 0.020), range of motion (2.11, p < 0.039), WOMAC (2.09, p < 0.04), and two-minute walk test (2.03, p < 0.046) showed improvement in functional ability.

Conclusion

The findings of this study showed that both groups showed improvement following treatment, but that the use of a KPD in combination with strengthening and balance retraining is more efficient in reducing pain and enhancing quality of life in patients with grade 1 or 2 knee OA than conventional CBT.

## Introduction

One of the most enfeebling diseases that causes discomfort and functional impairment that affects quality of life is osteoarthritis (OA), also known as degenerative joint disease of the knee [1]. It predominantly affects the knee joint, which plays a major role in supporting the body in an erect posture [2,3]. Stiffness, pain, and disability result from the worsening of joint cartilage and sclerosis of the bone under the cartilage's surface [4]. It sporadically occurs in older age people, above the normal age for females of 45, but is more prevalent in males younger than 45 [5]. Knee joints are thought to be impacted by OA at a rate of 76% [6,7]. Joint pain, tenderness, restricted movement, crepitus, periodic effusion, and varying degrees of inflammation are the main symptoms of the disease [8].

The level of severity of OA is rated from grade 0 to 4 depending on radiographic findings and the Kallgren and Lawrence classification [9]. In 0-grade OA, there are no significant changes. Although an X-ray can't identify any damage in grade 1 OA, osteophytes can be a warning indication and people may feel pain or discomfort. In grade 2, X-rays may show a narrowing in joint space and osteophyte, and patients may experience stiff and painful joints, particularly after relaxing for a while. In grade 3, there is a moderate narrowing of the joint space followed by the growth of osteophytes, some sclerosis, and some abnormalities of the bony ends. With the growth of osteophytes and bony end deformities in grade 4, joint space has significantly decreased. Stiffness is brought on by peri-articular, intra-muscular, and intra-articular adhesions [10]. The conservative method of care includes self-management training, exercise therapy, weight loss, knee braces, and analgesics [11].

A non-pharmacological treatment alternative for OA pain and stiffness reduction is contrast bath therapy (CBT). In this method, each limb of the body is immersed in warm water and then immediately immersed in cold water. Several times, this cycle is repeated. Furthermore, this can be used to alleviate swelling [12]. Warm water immersion increases blood flow and vasodilation, which can stimulate the production of endorphins and encephalin substances that relieve pain and increase comfort by blocking the transfer of pain signals [13]. Cold water immersion promotes vasoconstriction, which can lessen edema and relieve pain by reducing the pressure on pain receptors [13].

The knee pad device (KPD) based on vibration and alternate heating and cooling by using an ice pack is used to treat pain. The heating pads inside this device are designed to provide a temperature of 40-45°C. It is feasible to alter the heating and vibration modes according to the need. It is loaded with a 3000 mAh battery which can withstand 6-7 hours.

Through vibrations of molecules, infrared releases heat and can enter the skin up to 2-3 cm without irritating the outer layer of the skin. Heating enhances the tissues' supply of nutrients, blood flow, and intra-cellular fluid as well as removing metabolic waste. This results in decreased pain and muscle spasms. The use of an ice pack during cryotherapy enhances the threshold for pain and reduces its intensity [14].

The most frequently prescribed treatment for knee OA is likely to be exercise therapy. Numerous treatment guidelines endorse exercise therapy since the evidence suggests that it is beneficial for people with knee OA, even though its effects are limited [15].

Exercise therapy is the most often used method of managing OA of the knee. Land-based exercise programs have shown good effects on pain and life quality. In people with knee OA, proprioceptive exercises can increase proprioceptive acuity, reduce discomfort, and improve functional impairment in both weight-bearing and non-weight-bearing positions [14]. Otago exercise's main objective is to assist OA patients in enhancing their static as well as dynamic equilibrium through targeted limb strength and balance function retraining. The initial component of this exercise program consists of flexibility training, lower extremity muscular strength training, and balance function training. Walking is the second portion of the program [16].

## Materials and methods

At the musculoskeletal outpatient department of the Acharya Vinoba Bhave Rural Hospital and Ravi Nair Physiotherapy College, Wardha, India, a randomized controlled trial was carried out. Approval was obtained from the Institutional Ethical Committee of Datta Meghe Institute of Higher Education & Research (Deemed to Be University) with Ref. No. DMIMS(DU)/IEC/2022/797 and Clinical Trial Registration of India (CTRI/2022/05/042506). The research protocol is published in the Journal of Clinical and Diagnostic Research, before initiating the study. The study only included participants who were permitted to participate. A total of 60 people were included through simple random sampling and sorted into group A and group B using the sequentially numbered opaque sealed envelope method.

Under the supervision of a professor in the Department of Musculoskeletal Physiotherapy, the primary researcher, a postgraduate resident in physiotherapy, completed the randomization and allocation. A postgraduate resident in physiotherapy with the same level of experience who was aware of the trial and blinded to the intervention evaluated outcomes before the beginning of the research and just after it was completed.

Inclusion and exclusion criteria

**Figure 1:**
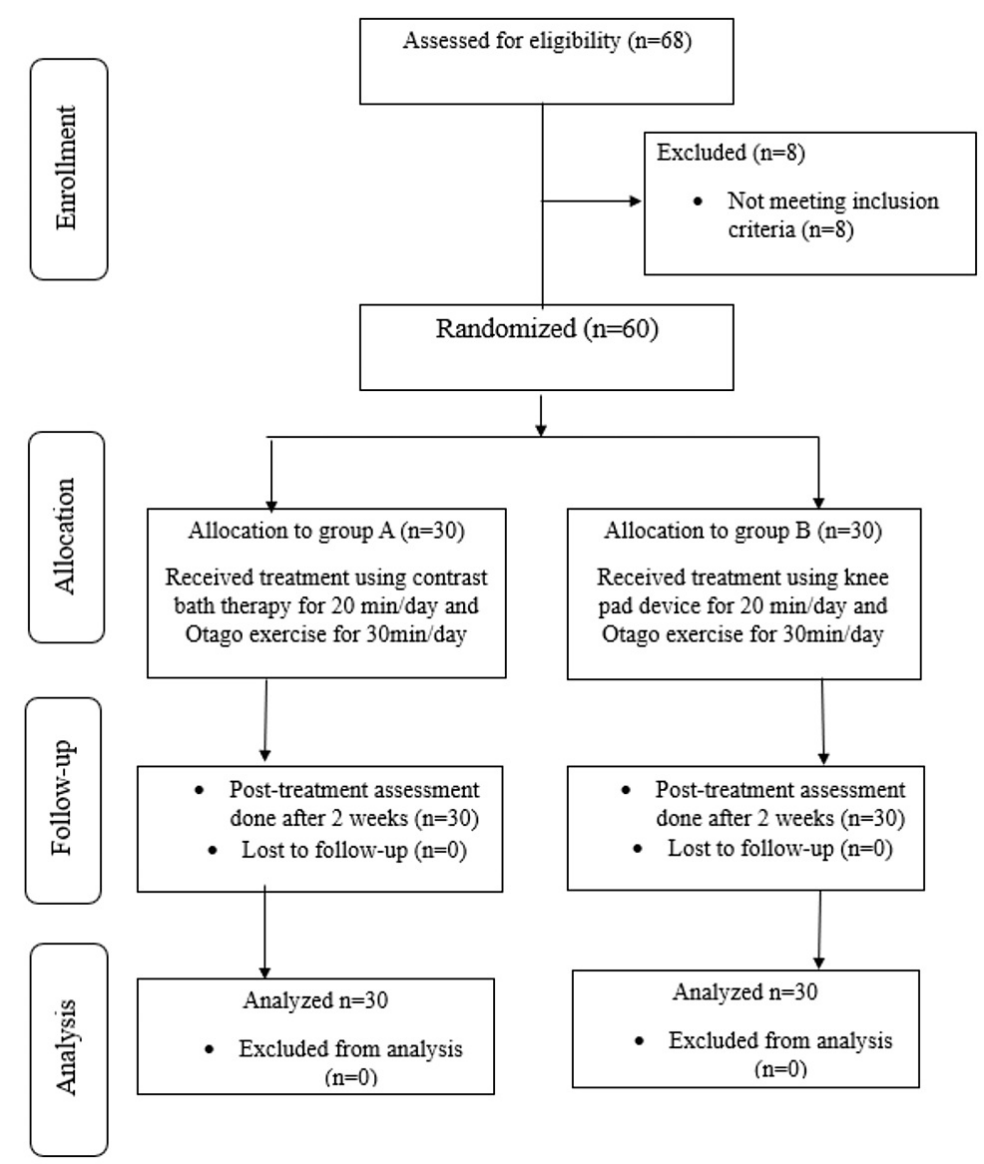
Methodology and progress of the research

Patients both male and female having age groups between 40 and 60 years, grade 1 or 2 unilateral knee OA, and suffering from stiffness less than 30 minutes were included in the study. Patients having grade 3 or 4 OA, superficial or deep sensory impairments, systemic illnesses, patients with severe disabilities, participating in other interventional studies, and not willing to participate were excluded from this study. Each participant gave their written, informed consent. The methodology of the research is illustrated in a flowchart in Figure 1.

Procedure

Group A received treatment with CBT. It was given in a sitting position, with the lower limb immersed in water up to the knee. The water in two immersion baths was adjusted to a temperature of 12˚C-14˚C and 38˚C-40˚C, respectively. The lower limb was soaked in warm water for four minutes, and then for one minute in cold water. The cycle was repeated for a total of 20 minutes, beginning with a warm water immersion.

**Figure 2:**
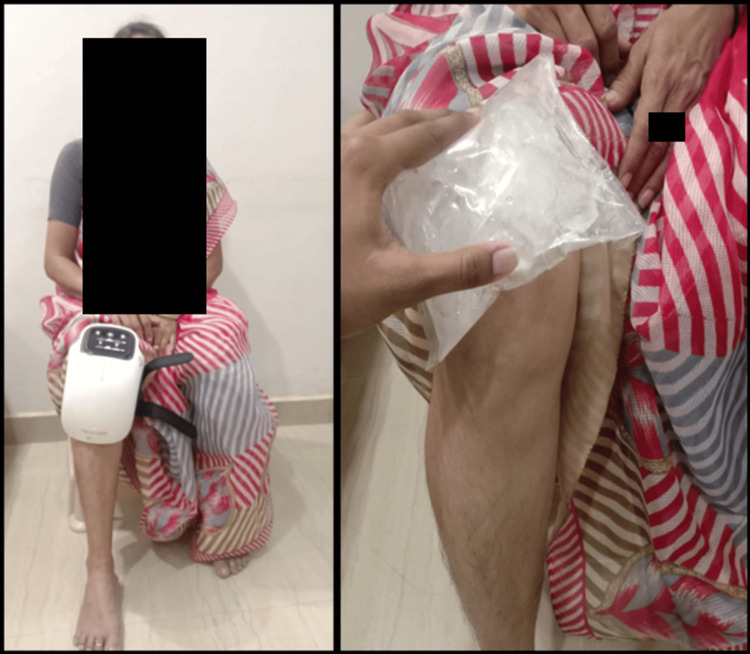
KPD application over the right knee and cryotherapy given with an ice pack KPD: Knee pad device

Group B received treatment with a KPD. Over the knee joint, this device was placed. Depending on the patient's needs, the heating and vibration mode was altered. After four minutes of application, this was followed by one minute of cryotherapy using an ice pack. For 20 minutes, this therapy was given as shown in Figure 2.

In both groups, the Otago exercise program (OEP) was carried out. The OEP takes about 20-30 minutes to complete. It starts with a gentle five-minute warm-up that includes flexibility exercises, then moves into lower-limb strengthening and balancing drills. Exercises to strengthen the knee extensors and flexors, the hip abductor, the ankle plantar flexors, and the dorsiflexors were performed. Resistance was provided by ankle weight cuffs with 10 repetitions in one set, three sessions per week for two weeks. In this program, balance retraining exercises included knee bending, walking and turning around on a figure-of-eight mark, sideways walking, backward walking, tandem stance, tandem walking, and one-leg stance. About 10 repetitions in one set, of each balance retraining exercise, were given for three sessions per week for two weeks.

Visual analog scale (VAS), Knee flexion range, Western Ontario and McMaster Universities Arthritis Index (WOMAC) scale, and distance covered in a two-minute walk test were taken as outcome measures pre and post-treatment.

Statistical analysis

The level of significance for the statistical analysis was set at p < 0.05, and descriptive and inferential statistics were calculated using the chi-square test, student-paired and unpaired t-tests, and the software used was IBM SPSS Statistics for Windows, Version 27 (Released 2020; IBM Corp., Armonk, New York, United States) and GraphPad Prism 7.0 (GraphPad Software, Boston, USA).

## Results

Participants were enrolled in all 68, and each participant's eligibility was determined. Of those, 8 failed to fulfill the criteria for inclusion. Groups A and B, each with 30 patients, were made up of 60 patients who matched the study's criteria. Patients in both groups were 40-60 years old. With a gender distribution of 9:7 males and 21:23 females in groups B and A, respectively. Along with this, 9:11 samples with grade 1 OA and 21:19 samples with grade 2 OA were seen in groups B and A, respectively. There was no statistically significant disparity in the patient ages, gender, or grade of OA between the two groups, according to the chi-square test results. Table 1 shows the subjects' baseline characteristics.

**Table 1:** Baseline characteristics The data in the above table shows the mean ± SD values of age, percentage of male and female ratio, and grade 1 and grade 2 OA ratio in both groups. p < 0.05 is considered as significant. NS: Not Significant; OA: Osteoarthritis

Baseline characteristics	Group A (control group)	Group B (intervention group)	p-value
Age in years	50.06 ± 6.66	51.43 ± 4.88	0.19, NS
Age range	40-60 yrs	40-60 yrs	
Gender			
Male	7 (23.33%)	9 (30%)	0.55, NS
Female	23 (76.67%)	21 (70%)	
Grade of OA			
Grade 1	11 (36.7%)	9 (30%)	0.79, NS
Grade 2	19 (63.3%)	21 (70%)	

Table 2 shows the statistical analysis of all the outcome measures and the significant values pre and post-treatment within groups and post-treatment between groups. The results of the study revealed that group B significantly outperformed group A in terms of pain, range of movement, WOMAC score, and the two-minute walk test.

**Table 2:** Outcome measures The above table shows the mean ± SD and p-values of all the outcome measures. Shows mean of VAS, knee flexion range, WOMAC score, and two-minute walk test pre and post-treatment of group A and group B and between-group analysis. p < 0.05 is considered to be significant. VAS: Visual analog scale; WOMAC: Western Ontario and McMaster Universities Osteoarthritis Index

Outcome measure	Group A (control group)		p-value	Group B (intervention group)		p-value	Mean difference (X ± SD)		p-value
	Pre-treatment	Post-treatment		Pre-treatment	Post-treatment		Group A	Group B	
VAS	7.37 ± 0.54	6.72 ± 0.80	0.0001	7.09 ± 0.69	5.95 ± 1.12	0.0001	0.66 ± 0.63	1.14 ± 0.88	0.020
Knee flexion range	100.90 ± 5.06	103.66 ± 5.14	0.0001	102.03 ± 6.76	106.96 ± 8.07	0.0001	2.76 ± 1.81	4.93 ± 5.31	0.039
WOMAC score	67.39 ± 3.70	64.44 ± 4.88	0.0001	66.97 ± 3.87	61.43 ± 6.69	0.0001	2.95 ± 3.47	5.53 ± 5.78	0.04
two-minute walk test	155.73 ± 19.33	169.43 ± 28.29	0.0001	156.33 ± 21.21	178.16 ± 24.15	0.0001	13.70 ± 15.76	21.83 ± 15.17	0.046

**Figure 3:**
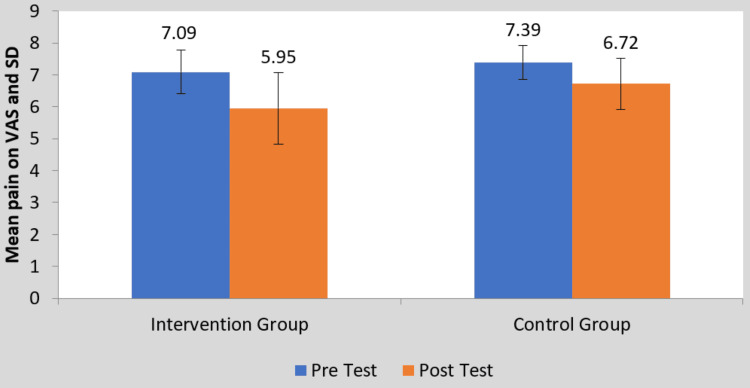
Comparison of VAS score between two groups pre and post-treatment VAS: Visual analog scale

The VAS score before and after treatment is shown in Figure 3. Both groups exhibited improvement after treatment, but group B, the interventional group showed significantly better outcomes. The findings of the student's unpaired t-test were significant (t-value = 2.39, p-value = 0.020) for the VAS between group analysis.

**Figure 4:**
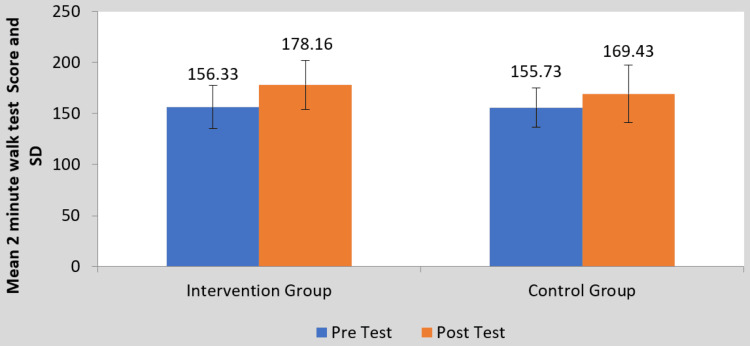
Comparison of two-minute walk test scores between two groups pre and post-treatment

Figure 4 shows the improvement in distance covered in the two-minute walk test in both groups, but the interventional group was better when compared with the control group. For between-group analysis, the student’s unpaired t-test was used, and the results came out to be significant (t-value = 2.03, p-value = 0.046) for group B.

## Discussion

In group A, CBT was given along with the OEP. Regarding pain, this group showed that many of them suffered from moderate pain which, post-treatment showed significant improvement. Similarly, a study by Abd EL Fatah et al., stated that contrast hydrotherapy effectively reduces pain and improves quality of life [17]. Another study by Rajapriya, also stated that the group that received contrast therapy showed more improvement than the group that only received treatment with heat application [18].

Endorphins and encephalin hormones, which help reduce pain, enhance comfort, and obstruct the transmission of pain input, are released when heat is applied. Applying cold reduces inflammation, which has an impact on pain inhibition, by causing vasoconstriction, which reduces cellulite permeability, cellular diffusion, and neutrophil migration [13].

In this study contrast therapy in both groups was given in the ratio of 4:1 which is a four-minute hot application and a one-minute cold application alternately. Similarly, a study by Shadgan et al. also used the ratio of 4:1 minute of hot-cold immersion for treatment and concluded that the contrast bath application improves oxygenation and tissue hemodynamics and supports its therapeutic effects [19].

Group B received treatment using a KPD along with the OEP. The device used in this study is self-operatable as it has a touched screen, displaying three modes of vibrations and heating which according to the patient’s requirement was managed. Similarly, a study by Priya et al., on individuals with knee pain using a smart knee pain relief pad which gives alternate heating and cooling along with vibrations, showed significant improvement in reducing pain. This device was connected to a smart smartphone to operate the intensity of heating, cooling, and vibrations according to the needs of the patients which was psychologically good for reducing pain. They could operate the required intensities on their own according to the pain [20].

This study showed improvement in scores on VAS post-treatment in both groups. Because physiological effect of heat therapy includes the reduction of pain, an increase in blood flow and metabolism, and an improvement in connective tissue elasticity [21]. Warming additionally accelerates the metabolism of the local tissue, which further aids in the healing process. The proven effectiveness of heat therapy for increasing the range of motion may be due to modifications in the viscoelastic characteristics of collagenous tissues brought on by heat [22].

A sympathetic vasoconstrictive reaction is triggered by the skin and muscle losing heat, which lowers blood circulation to the cooled tissues. A local anesthetic effect known as cold-induced neurapraxia results from the reduction of the activation threshold of tissue nociceptors and the conduction velocity of pain-signaling nerve signals during cold therapy [23,24]. A study by Shehata and Fareed showed that the effect of contrast therapy is greater when compared with individual heat or cold effects [25].

Both groups received contrast therapy, but group B additionally received a vibration effect along with alternate heat and cold treatment. When vibrations are administered locally, vibratory analgesia predominantly reflects a spinal mechanism, since signals from low-threshold mechanoreceptors suppress the activity of wide dynamic range neurons in the dorsal horn, which transmit information about stimulus pain to the brain. This helps to block the pain sensations [26]. Because of this physiological effect of vibrations along with contrast therapy, group B showed more improvement in pain score on VAS as compared to group A.

In this study improvement in the WOMAC score of both the groups was seen because of improvement in pain, range, and stiffness. Heating improves the extensibility of the tissues. The alternate hot and cold application removes the metabolic waste that stimulates the nociceptors thus alleviating pain.

The OEP is also included in this study and was given in both groups. A study by Cederbom and Arkkukangas concluded that the OEP could be a suitable evidence-based pain management and fall prevention program [27]. One of the studies by Salekar and Khandale also concluded that OEP is effective in increasing strength and balance [28]. Concentric and eccentric contractions of muscles are a part of resistance training exercises. Exercises like resistance and endurance training are particularly helpful for OA of large joints, like the knee, in terms of pain and balance [29]. Many recent studies that are now available have found that retro-walking, also known as backward walking, can lessen knee OA patients' symptoms and is one of their kinetic options. Retro-walking, which is counter-sequential to forward walking and has a beneficial effect on limb balance, gait synergy, and lower limb proprioception, is a useful exercise [30]. A two-minute walk test is also used in this study to assess the functional capacity of the patients. A study by Gacto-Sánchez et al. confirms that a two-minute walk test is a practical evaluation tool in replacement of a six-minute walk test [31].

This study demonstrated that, when assessed using the VAS for pain, the range of knee flexion on a goniometer, functional impairments on the WOMAC, and two-minute walk test after treatment sessions, both groups exhibited significant improvement. When the means of the two groups were compared for all outcome measures, group B, which was treated with a KPD, significantly outperformed group A, which received CBT.

Since this was a time-limited trial, follow-up was not feasible, indicating that the effects over time were not examined and that no control group did not receive any treatment. In this study, patients having unilateral grade 1 or 2 OA were included. So, in the future the study can be performed in grade 3 or 4 OA, patients with bilateral knee OA, and another pain-reducing intervention can be added to this treatment.

## Conclusions

This study intended to find the effects of CBT and KPD along with OEP in patients with OA knee. Statistical analysis showed post-treatment improvement in all the outcomes that is VAS, range, WOMAC score, and two-minute walk test of both the groups. Group B showed better results when compared with group A.

The study concluded that both groups improved after treatment, but using a KPD combined with strengthening and balance retraining was found to be more effective than conventional CBT in reducing pain and improving functional ability in patients with grade 1 or 2 knee OA.

## References

[1] Briggs AM, Cross MJ, Hoy DG, Sànchez-Riera L, Blyth FM, Woolf AD, March L (2016). Musculoskeletal health conditions represent a global threat to healthy aging: a report for the 2015 World Health Organization World Report on ageing and health. Gerontologist.

[2] Nandanwar R, Uttamchandani S, Deshmukh M (2021). Physiotherapy rehabilitation in patient with bow leg deformity. J Med Pharm Allied Sci.

[3] Birelliwar A, Jaiswal S, Wadhokar O (2021). Medial meniscal tear. J Med Pharm Allied Sci.

[4] Chang G, Xia D, Chen C (2015). 7T MRI detects deterioration in subchondral bone microarchitecture in subjects with mild knee osteoarthritis as compared with healthy controls. J Magn Reson Imaging.

[5] Pal CP, Singh P, Chaturvedi S, Pruthi KK, Vij A (2016). Epidemiology of knee osteoarthritis in India and related factors. Indian J Orthop.

[6] Uysal F, Basaran S (2022). Knee osteoarthritis. Turk J Phys Med Rehabil.

[7] Palazzo C, Nguyen C, Lefevre-Colau MM, Rannou F, Poiraudeau S (2016). Risk factors and burden of osteoarthritis. Ann Phys Rehabil Med.

[8] Bhaskar A, Areekal B, Vasudevan B (2016). Osteoarthritis of knee and factors associated with it in middle aged women in a rural area of central Kerala, India. Int J Community Med Public Health.

[9] Kohn MD, Sassoon AA, Fernando ND (2016). Classifications in brief: Kellgren-Lawrence classification of osteoarthritis. Clin Orthop Relat Res.

[10] Jawade S, Vardharajulu G, Naidu N (2020). Comparison of effectiveness of hold‑relax technique and maitland’s mobilization in improving range of motion in posttraumatic stiffness of knee joint. J Datta Meghe Inst Med Sci Univ.

[11] Dries T, VA VA Windt JW, Akkerman W, Kluijtmans M, Janssen RP (2022). Effects of a semi-rigid knee brace on mobility and pain in people with knee osteoarthritis. J Rehabil Med Clin Commun.

[12] Sathyan J (2018). A study to compare the effectiveness of hot bath versus contrast bath on level of pain perception among patients with arthritis in selected hospital, Kanyakumari district. http://repository-tnmgrmu.ac.in/id/eprint/11728.

[13] Rusminingsih E, Agustina N, Wulan D (2020). The effectiveness of contrast bath to reduce joint pain in the elderly. Medisains.

[14] Kim J, Jung H, Yim J (2020). Effects of contrast therapy using infrared and cryotherapy as compared with contrast bath therapy on blood flow, muscle tone, and pain threshold in young healthy adults. Med Sci Monit.

[15] Adhama AI, Akindele MO, Ibrahim AA (2021). Effects of variable frequencies of kinesthesia, balance and agility exercise program in adults with knee osteoarthritis: study protocol for a randomized controlled trial. Trials.

[16] Xie C, Wang W, Pei J, Wang H, Lv H (2020). Effect of otago exercise on falls in patients with osteoarthritis: a protocol for systematic review and meta-analysis. Medicine (Baltimore).

[17] Abd EL Fatah M, Weheida S, Mekkawy M (2019). Effect of contrast hydrotherapy on pain intensity and quality of life outcomes for patients with knee osteoarthritis. Assiut Sci Nurs J.

[18] Rajapriya G (2016). Effect of hot application versus contrast therapy on knee related symptoms among patients with knee osteoarthritis in selected community area at Perambalur. http://repository-tnmgrmu.ac.in/id/eprint/2861.

[19] Shadgan B, Pakravan AH, Hoens A, Reid WD (2018). Contrast baths, intramuscular hemodynamics, and oxygenation as monitored by near-infrared spectroscopy. J Athl Train.

[20] Priya L, Vignesh V, Krishnan V, Ajeesh RP (2018). Design and development of a smart knee pain relief pad based on vibration and alternate heating and cooling treatments. Technol Health Care.

[21] Nadler S, Weingand K, Kruse R (2004). The physiologic basis and clinical applications of cryotherapy and thermotherapy for the pain practitioner. Pain Phys.

[22] Bleakley CM, Costello JT (2013). Do thermal agents affect range of movement and mechanical properties in soft tissues? A systematic review. Arch Phys Med Rehabil.

[23] Algafly AA, George KP (2007). The effect of cryotherapy on nerve conduction velocity, pain threshold and pain tolerance. Br J Sports Med.

[24] Malanga GA, Yan N, Stark J (2023). Mechanisms and efficacy of heat and cold therapies for musculoskeletal injury. Postgrad Med.

[25] Shehata A, Fareed M (2013). Effect of cold, warm or contrast therapy on controlling knee osteoarthritis associated problems. Int J Medical Sci.

[26] Hollins M, McDermott K, Harper D (2014). How does vibration reduce pain?. Perception.

[27] Cederbom S, Arkkukangas M (2019). Impact of the fall prevention Otago Exercise Programme on pain among community-dwelling older adults: a short- and long-term follow-up study. Clin Interv Aging.

[28] Salekar S, Khandale S (2019). Effect of otago exercise program on balance and risk of fall in community-dwelling individuals having knee osteoarthritis. Int J Adv Res Dev.

[29] Bielecki JE, Tadi P Therapeutic Exercise. StatPearls [Internet].

[30] Wu Y, Lei C, Huangfu Z, Sunzi K, Yang C (2020). Effect of backward walking training on knee osteoarthritis: protocol of a systematic review and meta-analysis. BMJ Open.

[31] Gacto-Sánchez M, Lozano-Meca JA, Lozano-Guadalajara JV, Montilla-Herrador J (2023). Concurrent validity of the 2-and 6-minute walk test in knee osteoarthritis. Knee.

